# The effect of breastmilk and saliva combinations on the *in vitro* growth of oral pathogenic and commensal microorganisms

**DOI:** 10.1038/s41598-018-33519-3

**Published:** 2018-10-11

**Authors:** E. L. Sweeney, S. S. Al-Shehri, D. M. Cowley, H. G. Liley, N. Bansal, B. G. Charles, P. N. Shaw, J. A. Duley, C. L. Knox

**Affiliations:** 1 The Institute of Health and Biomedical Innovation, Faculty of Health, School of Biomedical Sciences, Queensland University of Technology, Brisbane, 4059 Australia; 2 School of Pharmacy, PACE, The University of Queensland, St Lucia, 4072 Australia; 3 School of Applied Medical Science, Taif University, Taif, 21974 Saudi Arabia; 4 Mater Research Institute, The University of Queensland, Woolloongabba, 4102 Australia; 5 School of Agriculture and Food Science, The University of Queensland, St Lucia, 4072 Australia

## Abstract

Neonates are exposed to microbes *in utero* and at birth, thereby establishing their microbiota (healthy microbial colonisers). Previously, we reported significant differences in the neonatal oral microbiota of breast-fed and formula-fed babies after first discovering a primal metabolic mechanism that occurs when breastmilk (containing the enzyme xanthine oxidase) and neonatal saliva (containing highly elevated concentrations of the substrates for xanthine oxidase: xanthine and hypoxanthine). The interaction of neonatal saliva and breast milk releases antibacterial compounds including hydrogen peroxide, and regulates the growth of bacteria. Using a novel *in vitro* experimental approach, the current study compared the effects of this unique metabolic pathway on a range of bacterial species and determined the period of time that microbial growth was affected. We demonstrated that microbial growth was inhibited predominately, immediately and for up to 24 hr following breastmilk and saliva mixing; however, some microorganisms were able to recover and continue to grow following exposure to these micromolar amounts of hydrogen peroxide. Interestingly, growth inhibition was independent of whether the organisms possessed a catalase enzyme. This study further confirms that this is one mechanism that contributes to the significant differences in the neonatal oral microbiota of breast-fed and formula-fed babies.

## Introduction

The infant is first exposed to bacteria *in utero* from the placenta during gestation^1^, then, at the time of birth, is colonised with rich and diverse microorganisms from maternal sites^2^, including the maternal lower genital tract, faeces and/or skin^3,4^, from milk feeds as well as from the general environment^5^. The microorganisms contribute to the development of the neonatal oral microbiota that will then colonise the remainder of the gastrointestinal tract (GIT). The GIT microbiota has been shown to have numerous important roles in establishing the infant’s innate and adaptive immune system, conferring protection against attacks from pathogenic microbes^6^.

Previously, we have reported that there are substantial differences between the oral bacterial microbiota of breast-fed and formula-fed neonates^7^. Our group discovered that neonatal saliva contains high levels of the metabolites xanthine and hypoxanthine, which are substrates of xanthine oxidase (XO), an enzyme that is highly abundant in breast milk^8^. When neonatal saliva combines with breastmilk during feeding, hydrogen peroxide (H_2_O_2_) at concentrations up to 100 micromolar are generated, and this in turn activates the ‘lactoperoxidase system’ (LPO) in milk to further produce other reactive oxygen species (ROS) and reactive nitrogen species (RNS)^8^. The production of these ROS and RNS was confirmed to have *in vitro* antibacterial activity: we previously demonstrated that the growth of *Staphylococcus aureus* and *Salmonella* spp. was inhibited during *in vitro* experiments when these pathogenic bacteria were exposed to physiological mixtures of simulated neonatal saliva and human breastmilk, whereas the commensal bacteria *Lactobacillus* spp. and *Escherichia coli* were unaffected^8^. These novel studies were unique in analysing the growth of human oral bacteria under conditions replicating breastfeeding *in vitro*, in contrast to optimised growth studies using nutrient broth and other bacteriological media that contain undefined concentrations of nucleosides and bases that may confound the experimental results.

During the initial *in vitro* experiments that we conducted, we investigated the effects of breastmilk and saliva mixtures, which generate peroxide and ROS/RNS, on bacterial growth following seeding with low numbers (200 colony forming units [CFU]) of the four species above^8^. We also investigated the antibacterial activity of micromolar concentrations of hydrogen peroxide, followed by 24 hrs incubation. However, these experiments did not investigate different combinations of bacteria, nor the higher concentrations of microorganisms that are known to be present within the oral cavity of infants.

Therefore, the current study was designed to investigate the *in vitro* effect of physiological mixtures of breastmilk and saliva on the growth of ten microbial species, including high and low initial concentrations of microorganisms over time. We examined further the effect of breastmilk-saliva mixtures on the growth of different combinations of microorganisms. Finally, we examined the effects of varying hydrogen peroxide concentrations on catalase-positive versus catalase-negative microorganisms, which were incubated for 24 hr in nutrient broth, a non-defined medium.

## Results

### Bacteria are inhibited over time in breastmilk and saliva mixtures

**Figure 1:**
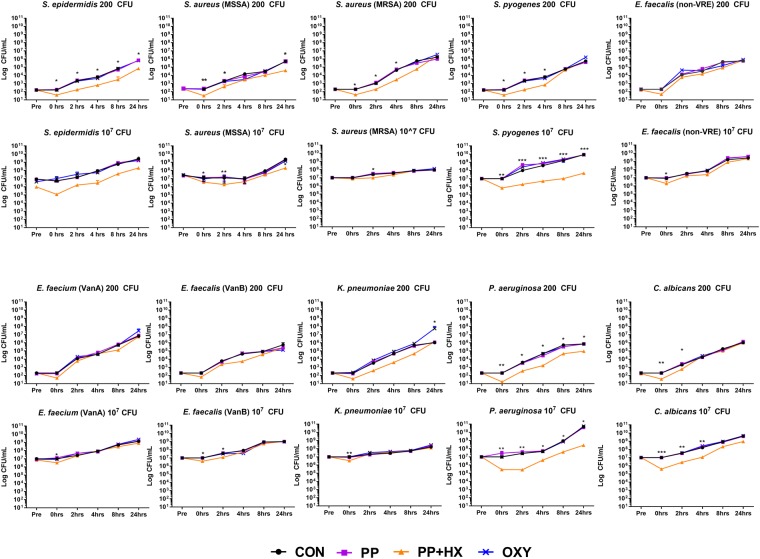
Microbial growth in the presence of human breastmilk and simulated neonatal saliva, seeded at 200 and 10^7^ CFU, then incubated at 37 °C for 24 hrs. All species grew in the control (CON) mixture of breastmilk and simulated neonatal saliva (Black line, circles). There was no benefit for most species when purine and pyrimidine bases/nucleosides (PP) were added (purple line, squares). However, when hypoxanthine and xanthine were also added (PP + HX), the growth of most microorganisms was immediately inhibited, due to the generation of oxidative radicals by the breastmilk xanthine oxidase and the lactoperoxidase system (orange line, triangles). Some bacteria were able to recover and continue to grow at rates similar to CON. When xanthine oxidase inhibitor oxypurinol (OXY) was added to the PP + HX mix, microbial growth was completely released from suppression by oxidative radicals (blue line, crosses). ^*^P < 0.05, ^**^P < 0.01, ^***^P < 0.001.

To demonstrate bacterial growth *in vitro* under conditions mimicking the *in vivo* environment of the infant mouth during breastfeeding, we exposed each of the ten microorganisms to saliva-breastmilk mixtures, and determined the viable numbers as CFU/mL over a time course of 24 hr and under four different experimental conditions that we described^8^. We found that the numbers of viable microorganisms were not initially affected when exposed to the control (CON) saliva and breastmilk mixture, and subsequently, growth ensued in most cases except for methicillin resistant *Staphylococcus aureus* (MRSA), 10^7^ CFU (black line, circles) (Fig. 1). Supplementation with purines and pyrimidines (PP) did not alter the growth patterns (purple line, squares). However, the numbers of viable microorganisms in each experiment decreased immediately when added to the PP + HX saliva-breastmilk mixture supplemented with hypoxanthine (H) and xanthine (X), and in many cases the growth did not recover to control levels following 24 hr incubation (orange line, triangles). The mixture of breastmilk containing xanthine oxidase and PP + HX saliva, which contains the substrates for xanthine oxidase, generates peroxide (and other oxidative radicals). The addition of the xanthine oxidase inhibitor oxypurinol (OXY) to the PP + HX mixture completely removed this suppression of growth (blue line, crosses).

For six of the ten of the microorganisms tested at low (200 CFU) concentrations (i.e. methicillin sensitive *Staphylococcus aureus* (MSSA), *S. aureus*, MRSA, *Staphylococcus epidermidis*, *Streptococcus pyogenes*, *Pseudomonas aeruginosa*, *Candida albicans*), and nine of the ten species (i.e. all except MRSA) at high (10^7^ CFU) concentrations after addition to the *in vitro* system, the decreases in viability were rapid (at 0 hrs) and significant in the PP + HX experimental group, but these decreases were not evident in the other saliva-breastmilk mixtures tested (Figure 1).

Interestingly, we also observed differences in the ability of the various microorganisms to ‘recover’ and grow in the presence of saliva-breastmilk mixtures over the remaining 24 hr of these experiments, which was highly dependent upon microorganism concentrations. For microorganisms seeded at high (10^7^ CFU) concentrations, including *S. pyogenes, Enterococcus faecalis* (VanB) and *C. albicans*, the inhibition of growth lasted for 2–4 hr, after which time microbial expansion/replication occurred rapidly. In general, microbial inhibition was more pronounced when larger numbers of microorganisms were seeded at the start of each experiment.

It was noteworthy that the inhibition of growth was unaffected by the presence or absence of a bacterial catalase enzyme, an enzyme that upon activation metabolises hydrogen peroxide. When the PP + HX saliva + microorganisms was mixed with milk the effects of the hydrogen peroxide was not neutralised. For species which do not possess catalase enzyme (*Streptococcus, Enterococcus* and *Candida* spp.) the extent of microbial inhibition during saliva-breastmilk incubation *in vitro* was similar for both starting concentrations of microorganisms (200 and 10^7^ CFU) and did not differ from catalase-positive (MRSA, MSSA, *S. epidermidis, Klebsiella pneumoniae, P. aeruginosa*) bacteria (Figure 1).

### Bacteria in mixtures exposed to breastmilk and saliva are observed to act independently in their inhibition by ROS

**Figure 2:**
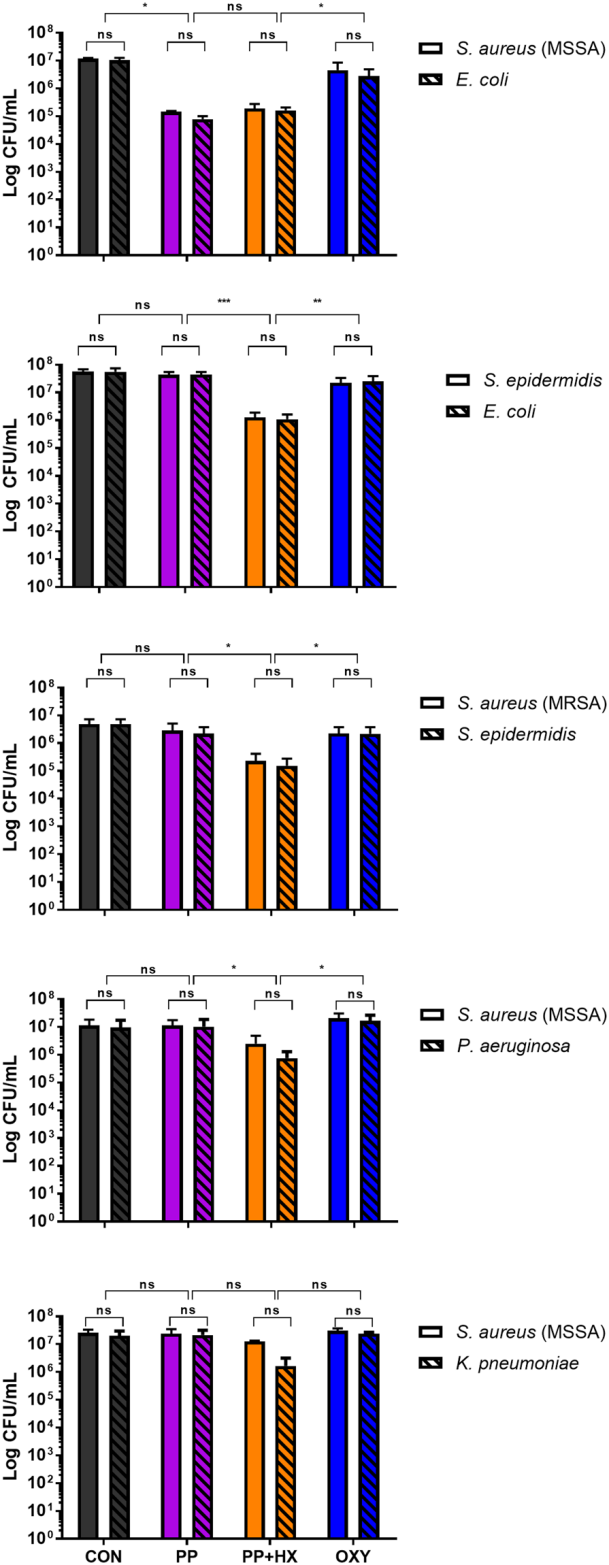
Mixtures of two microorganisms were exposed to human breastmilk and simulated neonatal saliva. The growth of both microorganisms remained similar over a 24 hr time period, irrespective of the ‘pathogenic’ or‘commensal’ nature of the organism within the infant oral cavity. ^*^P < 0.05, ^**^P < 0.01, ^***^P < 0.001.

When two species of bacteria were co-incubated in saliva-breastmilk mixtures, there were no significant differences in the numbers of microorganisms at the end of these 24 hr experiments, and remarkably these organisms did not appear to “compete” with one another but in fact adopted similar growth characteristics throughout the course of the experiment (Figure 2). In particular, the PP + HX saliva-breastmilk mixture, which generates hydrogen peroxide and reactive oxygen species, affected both microorganisms within the mixture in a similar fashion, regardless of whether they are considered to be “normal flora” or “pathogens”, and irrespective of whether these organisms possess a catalase enzyme.

### Bacteria were inhibited for prolonged periods by different concentrations of hydrogen peroxide

**Figure 3:**
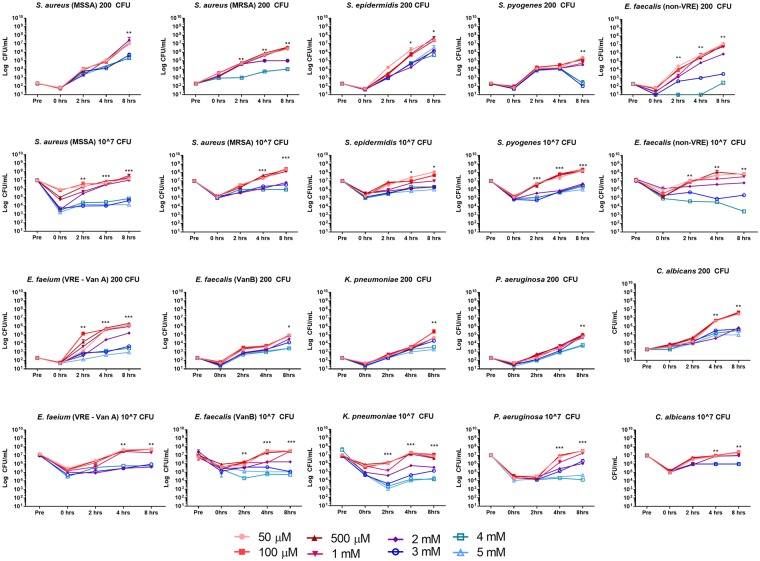
Effect of hydrogen peroxide concentrations on growth of ten bacterial species. Nutrient broth supplemented with varying concentrations of hydrogen peroxide (50 µM – 1 mM, pink-red lines) were seeded with either 200 or 10^7^ CFU. Most of the species exposed to low concentrations of hydrogen peroxide exhibited the ability to recover and grow exponentially, but some species were unable to recover when exposed to higher concentrations (2–5 mM, purple-blue lines) of hydrogen peroxide, over a 24 hr period. ^*^P < 0.05, ^**^P < 0.01, ^***^P < 0.001.

We further investigated whether at hydrogen peroxide concentrations of 5 μM – 5 mM, (added directly to incubations) was able to inhibit the growth of the ten microorganisms studied whilst incubated in nutrient broth, in a manner similar to that tested in our previous experiments with four microorganisms^8^. All ten microorganisms were affected in a concentration-dependent manner (Fig. 3). After an initial decrease in the CFU/mL, the Gram-positive organisms (MRSA, MSSA, *S. epidermidis, S. pyogenes* and *Enterococcus* spp.) and the yeast *C. albicans* were able to recover and achieve logarithmic growth in the presence of up to 2 mM hydrogen peroxide. However, peroxide concentrations above 2 mM inhibited the Gram-positive bacterial growth significantly, and the growth did not recover nor achieve logarithmic growth at the later time points (Fig. 3). Gram-negative microorganisms (*K. pneumoniae, P. aeruginosa*) were able to recover and grow in slightly higher concentrations of hydrogen peroxide (up to 3 mM), but were frequently unable to recover and reach logarithmic growth when exposed to peroxide concentrations above 3 mM.

## Discussion

The composition of the neonatal oral microbiota is an important factor in human health and wellbeing; changes to these bacterial communities are likely to have important implications for infection or disease early in life^2,6^. While it is reported that the adult oral microbiota is stable^9^, we have demonstrated previously that the neonatal oral microbiota is much more dynamic and may be altered according to the mode of feeding within the first few months of life^7^. We have also shown previously, using an *in vitro* culture system, that the growth of some key microorganisms is altered by the release of micromolar concentrations of hydrogen peroxide that are generated when breastmilk and simulated neonatal saliva are combined, modelling the *in vivo* environment in the infant mouth during breastfeeding^8^. Here, we present further *in vitro* evidence that the low concentrations of H_2_O_2_ and ROS generated metabolically are likely to affect the growth of microorganisms in the infant’s mouth over time.

For many of the microorganisms tested in these experiments, we demonstrated that after they were exposed to supplemented neonatal saliva containing xanthine oxidase substrates (PP + HX) in combination with breastmilk, there was an immediate reduction in the numbers of viable organisms. By contrast, this decrease in viable numbers was not observed when the microorganisms were exposed to supplemented neonatal saliva that contained oxypurinol (OXY), the active form of allopurinol, which inhibits the xanthine oxidase enzyme present in breastmilk. These observations are consistent with the mode of action of the metabolic pathway previously reported by us^8^. When neonatal saliva, containing high concentrations of xanthine and hypoxanthine, combines with breast milk containing xanthine oxidase, hydrogen peroxide, a key oxidative radical is released as a metabolic end-product. As an oxidative biocide, hydrogen peroxide attacks cells by damaging thiol groups in proteins and enzymes as well as by oxidising cell membrane lipids; these ultimately affect cell wall integrity and permeability^10^. It is well known that some bacteria have evolved defence mechanisms to protect themselves against the effects of hydrogen peroxide, such as pigmented bacteria (e.g. *Prevotella* and *Porphyromonas* spp. both members of the Phylum *Bacteroidetes*) containing cell surface layers of ferrihaems which may destroy hydrogen peroxide^11^, as well as bacteria which possess the catalase enzyme^12^. Interestingly, we reported previously that the genus *Prevotella* was significantly more abundant in the microbiota of mouths of formula-fed infants compared to breast-fed infants^7^. The ability of the surface layers of ferrihaems of *Prevotella* spp. to degrade hydrogen peroxide may account for these differences and for the higher prevalence of Phylum *Bacteroidetes* in the mouths of formula-fed neonates compared to breast-fed neonates^7^. However, the micromolar concentrations of hydrogen peroxide released by the LPO metabolic activity would be predicted as insufficient to activate the prokaryotic catalase enzyme in the microorganisms tested within this study.

We observed no significant differences in the inhibition of growth in the presence of saliva that was supplemented with xanthine and hypoxanthine (PP + HX) in organisms that were catalase-positive, compared to organisms that did not possess a catalase enzyme. This trend was consistent over the course of each 24 hr experiment. Previous studies investigating the kinetics of the prokaryotic catalase enzyme found that, depending on the microbe in question, the activation constant (Km) of the catalase enzyme can vary between 60–1000 µM^13^. Furthermore, as observed within our previous study^8^, fresh breastmilk contains a median hydrogen peroxide concentration of 27 µM, and the mixing of breastmilk and neonatal saliva results in the production of an additional 40 μM of H_2_O_2_. The combined effect of approximately 67 µM of hydrogen peroxide was shown to reduce the CFU of the microorganisms we tested by up to 10^1^ CFU, as demonstrated within this current study (see Figure 1 & Summary Table 1). Interestingly, this micromolar range coincides with estimates of the peroxide concentrations present within cells and wounds^14^. The activity of hydrogen peroxide on these organisms was further confirmed as this significant reduction in growth did not occur when the enzyme xanthine oxidase was inhibited by oxypurinol (OXY), and when the substrates xanthine and hypoxanthine were not present within the saliva (PP and CON) (Figures 1 and 2).

Additionally, we did not observe any correlations between the period of time that bacterial growth was suppressed and the ‘commensal’ or ‘pathogenic’ nature of the microorganisms studied. Microorganisms considered to be pathogens when found in the infant oral cavity, including *K. pneumoniae* and *P. aeruginosa*, showed no significant differences in their ability to recover and continue to grow exponentially, when compared to organisms that would be considered as normal flora of the oral cavity, including *S. aureus* and *S. epidermidis* (Figures 1 and 3). We also identified an interesting trend when co-incubating multiple microbial species in saliva-breastmilk mixtures. We hypothesised initially that the growth of one microbial species may predominate over another when placed into our *in vitro* culture system. However, we observed the opposite; the number of CFUs for each of the bacterial species exposed in combination (to the supplemented neonatal saliva mixture and breast milk; PP + HX) decreased by approximately 10^1^ – 10^2^, with the microbial growth patterns tending to be similar to one another, rather than a single organism’s growth predominating at 24 hrs of co-incubation. We hypothesise that this change in growth behaviour may arise from communication between these organisms during *in vitro* incubations, as other studies have reported the presence of quorum sensing molecules within human breast tissue^15^, which is consistent with the ability of microorganisms to communicate at this site.

Our study has demonstrated a proven role for hydrogen peroxide in regulating microbial growth, as well as a potential role for ROS and RNS generated when the lactoperoxidase system is activated. While these findings are novel, these are not the only known antimicrobial compounds which may affect the growth of microorganisms in the oral cavity of neonates. Breastmilk contains complex sugars, such as human colostrum hexasaccharide that has been shown to degrade the well recognised quorum sensing molecule acyl-homoserine lactones (AHL). Further experiments demonstrated that degradation of AHLs significantly reduced the expression of bacterial virulence factors and reduced the generation of antibiotic resistance in *S. aureus*^16^. Similarly, the breastmilk oligosaccharides 2′-fucosyllactose and 6′-sialyllactose have been shown to exert negative effects on *E. coli* adherence to Caco-2 human colon cells^17^, suggesting that these breastmilk components may play a role in preventing pathogenic microorganisms from colonising the infant gut. Secreted immunoglobulin (Ig) A, a mucosal-targeted immunoglobulin is also present in high concentrations in breastmilk and can be found in large amounts within infant faeces during the first few months of breastfeeding, thereby potentially playing a crucial role in shaping the infant oral and gut microflora^18^. Human breastmilk is also high in lysozymes that degrade glycans in the bacterial cell wall^18^. Lactoferrin, an iron-binding glycoprotein, is also abundant within human breastmilk and infant saliva and is known to exhibit both bacteriostatic and bactericidal activities^18^, with studies showing that prophylactic administration of lactoferrin reduces the incidence of late-onset sepsis in premature infants^19^. These studies, along with our own findings that oxidative radicals can regulate the oral microbiota, support the important role that breastfeeding plays during infancy and further expands our own knowledge of the role beyond nutrition that breastfeeding may have in regulating the oral and gastrointestinal microflora. These studies also suggest that pathogenic microorganisms may be ‘disarmed’ of their virulence traits, which may help to explain why some of the organisms we tested in our experiments containing mixtures of microorganisms do not compete with one another but appear, instead, to grow in synergy.

Our finding that microorganisms were inhibited for up to 24 hr following incubation with breastmilk and saliva is novel; it was, however, not unexpected. A previous study by our group demonstrated that the oral microbiome is dynamic between four – eight weeks of life and the oral microbiome differs as a consequence of feeding^7^. The phyla *Firmicutes* and *Actinobacteria* were the predominant bacteria present in the mouths of babies who were exclusively breastfed or fed formula milk respectively. However, for infants who were exclusively breastfed, bacteria of the phylum *Proteobacteria* were more abundant when compared to the mouths of infants who were fed formula milk. The *Proteobacteria* are common bacteria in the GIT of neonates and are also the predominant phylum (65%) detected in human breastmilk. The phylum *Bacteriodetes* and genus *Prevotella* were significantly more likely to be found in the oral cavity of babies who were exclusively fed formula milk, compared to the oral microbiota of babies who were exclusively breastfed^7^. While this investigation of the *in vivo* oral microbiota demonstrated differences in the abundance of certain microbes, we should note that our *in vitro* experiments do not take into account the breadth of other factors (antimicrobial and environmental) that may further influence the growth of microorganisms. However, taken together, our experiments show that breastmilk and saliva mixtures are able to differentially alter the growth of microorganisms, either alone or in combination with other factors.

In this novel study we have attempted to mimic the *in vivo* environment of the neonatal mouth during breastfeeding. The combination of breastmilk with saliva containing hypoxanthine and xanthine, generates hydrogen peroxide, which activates the lactoperoxidase system and results in reduced bacterial numbers. We also showed that microorganisms incubated in nutrient broth (which also contains nucleosides and bases), titrated with varying concentrations of hydrogen peroxide, altered the growth of microorganisms over time (Fig. 3), thereby confirming independently the antibacterial role of micromolar concentrations of hydrogen peroxide.

The hydrogen peroxide, as well as ROS and RNS, produced when breastmilk and saliva combine, will then be swallowed and subjected to the neonatal stomach secretions. It is well-established that the neonatal GIT microbiota varies according to the mode of feeding^20–23^. It is encouraging that a recent study^24^ demonstrated the presence of bioactive hydrogen peroxide within the rat neonatal stomach 2 hr post feeding. This confirms that peroxide derived from breastmilk and/or saliva remains active at low pH (pH = 4; similar to the neonatal stomach and GIT) and therefore this oxidative radical may continue to regulate the microbiota within the infant GIT. Therefore, we hypothesise that the swallowing of breastmilk and saliva mixtures may have complementary and/or synergistic effects with intra-gastric fluids and that these combined mixtures will further regulate the stomach microbiota.

In conclusion, this current research further expands our understanding of the metabolic pathway that is activated when breastmilk and saliva combine *in vivo*, further supporting a role for micromolar concentrations of hydrogen peroxide in regulating the growth of microorganisms in the neonatal mouth and gut. The study adds to the body of evidence that breastfeeding plays more than a simple nutritional function during infancy, and expands the evidence that breast-milk saliva interactions play an important role in the establishment of the infant’s microbiota. This may have important implications in settings where the normal interaction of breast milk and saliva is precluded, such as during intragastric tube feedings for infants who are preterm or have upper gastrointestinal malformations. Studies that investigate and compare concurrently differences in the neonatal oral microbiota and the gut microbiota may further elucidate the importance of breast-feeding versus formula-feeding in establishing the infant microbiota.

## Methods

### Ethical Clearance and specimen collection

Adult saliva and expressed breastmilk were collected from donors after written informed consent was obtained. The research was approved by the Human Research Ethics Committees of the University of Queensland (Reference: 2011000388) and Mater Health Services (Reference: 1652M and 2012_01LNR). All specimens were collected according to the relevant guidelines set out by the University of Queensland and Mater Health Services. Written informed consent was obtained from all patients prior to the collection of these specimens.

Adult saliva was pooled, heat-inactivated at 56 °C, centrifuged, filtered (0.2 µm, to remove bacteria and salivary and mucosal cells) and supplemented with concentrations of purine and pyrimidine nucleosides/bases resembling those found in human infant saliva, as previously described^8^. Similarly, breastmilk samples from 24 mothers were pooled and prepared as previously described^8^.

### Preparation of microorganisms for growth experiments

*S. epidermidis* (ATCC 14990), *S. aureus* (ATCC 29213; methicillin-sensitive; MSSA), *S. aureus* (ATCC 33591; methicillin-resistant; MRSA), *S. pyogenes* (clinical isolate), *E. faecalis* (clinical isolate; vancomycin-sensitive), *E. faecium* (clinical isolate; vancomycin-resistant; VanA), *E. faecalis* (clinical isolate; vancomycin- and teicoplanin-resistant; VanB), *P. aeruginosa* (ATCC 27853), *K. pneumoniae* (ATCC 27736), *E. coli* (clinical isolate) and *C. albicans* (clinical isolate) were obtained from the microbial culture collection at the Queensland University of Technology (QUT; Brisbane, Australia). Bacteria were cultivated in nutrient broth (Oxoid Pty. Ltd., Australia) until logarithmic growth was achieved, then the microorganisms were pelleted by centrifugation, resuspended in 10% (v/v) glycerol, and aliquoted and stored at −80 °C prior to use. An aliquot was thawed and quantified by serial dilution followed by determination of viability as the number of colony-forming units (CFU) per millilitre of stock solution. We then performed experiments with both low (200 CFU) and high (10^7^ CFU) numbers of microorganisms (Figures 1 and 3). This was done in order to simulate a typical number of microorganisms that would be ingested and may result in an infection, or high (10^7^ CFU) numbers of microorganisms that would be consistent with the amount of oral microflora in the oral cavity of neonates.

The microorganisms were selected to represent a range of Gram-positive, Gram-negative, catalase-positive and catalase-negative microorganisms that are representative ‘commensal’ (normal regional flora) and ‘pathogenic’ organisms within the neonatal oral cavity. These organisms also exemplify some of the most common phyla that we previously observed in the mouths of breast-fed and formula-fed neonates during a previous study^7^.

### *In vitro* analyses of bacterial growth in the presence of breastmilk-saliva mixtures

Assays were conducted as previously described^8^, with some minor modifications. Briefly, assays were carried out in flat-bottom 96-well microtiter plates (ThermoFisher Scientific, Australia). Thawed stocks of each microorganism were diluted in sterile phosphate-buffered saline (PBS; 0.9%) to a final concentration of 200 CFU or 10^7^ CFU. The microtiter plate wells for each experiment contained: 25 µL of breastmilk; 25 µL of bacterial suspension (at 200 CFU or 10^7^ CFU final concentrations); and 50 µL of four different simulated neonatal saliva formulations^8^ comprising: CON (control) = saliva with no supplements; PP = saliva supplemented with the purine and pyrimidine bases/nucleosides (11 μM inosine, 12 μM adenosine, 7 μM guanosine, 5.3 μM uracil, 12 μM uridine, which are the median concentrations found in neonatal saliva; PP + HX = PP further supplemented with xanthine oxidase substrates, 27 μM hypoxanthine (H) and 20 μM xanthine (X); and OXY = PP + HX with the addition of 100 μM oxypurinol, an inhibitor of xanthine oxidase. The microtiter plates were sealed with sterile film (ThermoFisher Scientific, Australia) and incubated at 37 °C, O_2_ for 24 hrs with mixing (200 rpm). Aliquots of each well were then serially diluted, inoculated onto the surface of nutrient agar plates and incubated (37 °C, O_2_, 24 hr) after which time colonies were counted, in order to determine the final number of colony-forming units (CFU)/mL in each well. For each microorganism the growth experiments were repeated in triplicate, a total of three times.

### Growth kinetics of microorganisms in the presence of breastmilk-saliva mixtures

Growth kinetics assays were prepared as per the above protocol, in 96-well microtiter plates. The numbers of microorganisms were quantified immediately prior to their addition to simulated saliva and breastmilk (which was termed “pre-inoculation”) and immediately after addition of breastmilk-saliva with each microbe (t = 0 hrs). The plates were then sealed with sterile film and incubated (as above); aliquots were taken subsequently at the time points of 2 hrs, 4 hrs, 8 hrs and 24 hrs. For each microorganism, a separate plate was used for each time point, so as not to disrupt the growth patterns during the removal of plates from the incubator for sampling. At each time point, an aliquot was serially diluted and each dilution was further sub-cultured onto the surface of an agar plate. The CFU/mL of each well was determined as described above.

### Growth of mixed cultures of microorganisms in the presence of breastmilk-saliva mixtures

We also conducted experiments in which two species were added together for *in vitro* growth experiments, to investigate the effects of saliva/breastmilk mixtures on each microbial species when co-incubated. For these experiments, two organisms (200 CFU of each microbe) were added together with breastmilk and saliva mixtures, as outlined above. The growth incubation conditions and non-selective media were used as described in the above section *in vitro* analyses of bacterial growth. Morphological differences in both organisms were used to differentiate the number of colonies of each microorganisms added to the experiment, no selective media was used.

### Hydrogen peroxide concentrations and the effects on catalase-positive and catalase-negative microorganisms

To determine the effects of hydrogen peroxide on catalase-positive and catalase-negative microorganisms, bacteria were diluted to a final concentration of 200 CFU or 10^7^ CFU in sterile PBS. For each experiment, a 75 µL aliquot of sterile nutrient broth (Oxoid Pty Ltd, Australia; which does not contain hydrogen peroxide) was inoculated with sterile hydrogen peroxide at final concentrations of 50 µM, 100 µM, 500 µM, 1 mM, 2 mM, 3 mM, 4 mM or 5 mM, in triplicate, within a 96-well microtiter plate (ThermoFisher Scientific). The numbers of microorganisms were quantified immediately prior to the addition (“pre-inoculation”) and immediately following the addition of bacteria to the H_2_O_2_-supplemented nutrient broth (t = 0 hrs). The plate was then sealed and incubated (as above). Subsequently aliquots from each well were collected at the time points specified above. The number of bacterial CFU/mL in each well, for each concentration of H_2_O_2_, then was determined using serial dilutions and sub-culturing onto the surface of nutrient agar plates as described above in order to determine the concentrations of hydrogen peroxide that were capable of inhibiting the growth of catalase-positive and catalase-negative microorganisms.

### Statistical analysis

For all *in vitro* experiments, analysis of variance (ANOVA) with repeated measures was used to determine statistical significance for each experimental group over time. Analyses and figures were conducted using GraphPad Prism (v7) and statistical significance was accepted as *P* < 0.05.

## Electronic supplementary material

Supplementary Table 1
