# Supplementary material for: The effect of breastmilk and saliva combinations on the in vitro growth of oral pathogenic and commensal microorganisms
Source: Sci Rep. 2018 Oct 11;8:15112. doi: 10.1038/s41598-018-33519-3 (PMC6181944; doi:10.1038/s41598-018-33519-3)
Supplement: Supplementary file 1 — Supplementary Table 1 [file 41598_2018_33519_MOESM1_ESM.docx]

**Title:** The effect of breastmilk and saliva combinations on the *in vitro* growth of oral pathogenic and commensal microorganisms

**Authors:** Sweeney EL^1*^, Al-Shehri SS^3^,Cowley DM^4^, Liley HG^4^, Bansal N^2,5^, Charles BG^2^, Shaw PN^2^, Duley JA ^2, 4†,^ Knox CL^1†^

^1^The Institute of Health and Biomedical Innovation, Faculty of Health, School of Biomedical Sciences, Queensland University of Technology, Brisbane, 4059, Australia

^2^ School of Pharmacy, PACE, The University of Queensland, St Lucia, 4072, Australia

^3^ School of Applied Medical Science, Taif University, Taif, 21974, Saudi Arabia

^4^Mater Research Institute, The University of Queensland, Woolloongabba, 4102, Australia

^5^ *School of Agriculture and Food Science, The University of Queensland, St Lucia, 4072, Australia*

** Corresponding author:* [*el.sweeney@qut.edu.au*](mailto:el.sweeney@qut.edu.au)

^†^ *Contributed equally to the paper*

**Supplementary Table 1.** Log differences in CFU/mL at 24 hours for CON and PP+HX experimental groups. Microbial growth in the presence of human breastmilk and simulated neonatal saliva with control saliva (CON) and saliva with bases/nucleosides, plus xanthine and hypoxanthine (PP+HX) were compared at 24 hrs after incubation. Key differences were observed, indicating that growth of some organisms were affected for up to 24 hrs.

**200 CFU starting concentration of microorganisms:**

| **Microorganism** | **Mean CFU/mL at 24 hrs PI - CON** | **Mean CFU/mL at 24 hrs PI – +PP** | **Log difference** |
| --- | --- | --- | --- |
| *S. epidermidis* | 7.3 x 10^5^ | 7 x 10^4^ | 1.0 |
| *S. aureus* (MSSA) | 5.3 x 10^5^ | 4 x 10^4^ | 1.1 |
| *S. aureus* (MRSA) | 1.7 x 10^6^ | 2.7 x 10^6^ | -0.2 |
| *S. pyogenes* | 5.7 x 10^5^ | 4.7 x 10^5^ | 1.1 |
| *E. faecalis* (non-VRE) | 7.2 x 10^5^ | 6 x 10^5^ | 0.08 |
| *E. faecium* (VanA) | 6.7 x 10^6^ | 5.5 x 10^6^ | 0.09 |
| *E. faecalis* (VanB) | 5.7 x 10^5^ | 2.3 x 10^5^ | 0.4 |
| *K. pneumoniae* | 1.1 x 10^6^ | 1.4 x 10^6^ | -0.1 |
| *P. aeruginosa* | 7.3 x 10^5^ | 9.3 x 10^4^ | 0.89 |
| *C. albicans* | 1.1 x 10^6^ | 1 x 10^6^ | 0.04 |

**10^7^ CFU starting concentration of microorganisms:**

| **Microorganism** | **Mean CFU/mL at 24 hrs PI - CON** | **Mean CFU/mL at 24 hrs PI – +PP** | **Log difference** |
| --- | --- | --- | --- |
| *S. epidermidis* | 2.7 x 10^9^ | 2 x 10^8^ | 1.1 |
| *S. aureus* (MSSA) | 2.4 x 10^9^ | 2 x 10^8^ | 1.1 |
| *S. aureus* (MRSA) | 9.3 x 10^7^ | 9.7 x 10^7^ | -0.2 |
| *S. pyogenes* | 8.7 x 10^9^ | 4.7 x 10^7^ | 2.3 |
| *E. faecalis* (non-VRE) | 2.7 x 10^9^ | 2.2 x 10^9^ | 0.09 |
| *E. faecium* (VanA) | 1.4 x 10^9^ | 8.3 x 10^8^ | 0.23 |
| *E. faecalis* (VanB) | 1 x 10^9^ | 1.1 x 10^9^ | -0.04 |
| *K. pneumoniae* | 1.7 x 10^8^ | 1.3 x 10^8^ | 0.1 |
| *P. aeruginosa* | 5 x 10^10^ | 3 x 10^8^ | 2.2 |
| *C. albicans* | 4.3 x 10^9^ | 9.3 x 10^8^ | 0.7 |
